# Association between objective measures and parent-reported measures of child tobacco smoke exposure: A secondary data analysis of four trials

**DOI:** 10.18332/tid/150296

**Published:** 2022-06-29

**Authors:** Michal Bitan, David M. Steinberg, Sandra R. Wilson, Amy E. Kalkbrenner, Bruce Lanphear, Melbourne F. Hovell, Vicki Myers Gamliel, Laura J. Rosen

**Affiliations:** 1 Department of Statistics and Operations Research, Tel Aviv University, Tel Aviv, Israel; 2 School of Computer Science, The College of Management Academic Studies, Rishon LeZion, Israel; 3 Department of Medicine, Stanford University School of Medicine, Palo Alto, United States; 4 Joseph J. Zilber School of Public Health, University of Wisconsin-Milwaukee, Milwaukee, United States; 5 Faculty of Health Sciences, Simon Fraser University, Vancouver, Canada; 6 School of Public Health, San Diego State University, San Diego, United States; 7 The Gertner Institute, Chaim Sheba Medical Center, Tel Hashomer, Ramat Gan, Israel; 8 Department of Health Promotion, Tel Aviv University, Tel Aviv, Israel

**Keywords:** tobacco smoke exposure, correlations, biomarkers, parental report

## Abstract

**INTRODUCTION:**

Tobacco smoke exposure (TSE) harms children and adults. Studies of childhood TSE exposure often relies on parental reports, but may benefit from objective measures. The objective of our study was to study the relationship between reported and objective measures of TSE.

**METHODS:**

We analyzed data from four intervention trials, conducted in clinical or community settings, to identify objective measures most closely associated with parent-reported measures and the optimal set of parent-reported measures for predicting objective measures. We also assessed whether there was a learning curve in reported exposure over time, and the importance of replicate biomarker measures.

**RESULTS:**

Correlations between objective and parent-reported measures of child TSE were modest at best, ranging from zero to 0.41. Serum cotinine and urinary cotinine were most strongly associated with parental reports. Parental questions most closely related to biomarkers were number of cigarettes and home smoking rules; together these formed the best set of predictive questions. No trial included all objective measures and all questions, precluding definitive statements about relative advantages. Within-subject repeatability of biomarker measures varied across studies, suggesting that direct pilot data are needed to assess the benefit of replicate measurements.

**CONCLUSIONS:**

Improvements in objective and parent-reported child exposure measurements are needed to accurately monitor child TSE, evaluate efforts to reduce such exposure, and better protect child health.

## INTRODUCTION

Childhood exposure to tobacco smoke harms children and adults^1-7^. According to the U.S. Surgeon General, tobacco smoke exposure (TSE) is a causal risk factor in a variety of child ailments, including sudden infant death syndrome, acute respiratory infection, middle-ear disease, early onset of wheeze illnesses, retardation of lung development, and decreased lung function in childhood^1^. Recently a causal relationship has been documented between long-term TSE in children and adverse cardiovascular outcomes in adulthood^8^.

Scientists have assessed TSE in children in various ways, including parental reports, monitoring of settled or airborne tobacco smoke in children’s homes, and objective biomarkers of TSE measured in blood, saliva, hair, nails, and urine^9^. Objective biomarkers, such as cotinine, are considered the most accurate way to assess an individual’s exposure. Biomarkers are, however, more expensive than querying parents and therefore are used less frequently. Many studies rely heavily on parental reports^10,11^. Understanding the relationship between specific questions and objective measures of children’s exposure could potentially contribute to more accurate assessment of exposure. Previous research has examined correlations between objective measures and parental reports^12-17^. To the best of our knowledge, however, no prior work has used actual field research to focus on the question of identifying the best set of parent-reported measures for predicting objective measures or to investigate the correlations between objective measures and parental reports over time and between groups.

Our primary goal was to understand the relationship between parent-reported TSE and objective measures of TSE. We used data from four randomized controlled trials^18-20^ which assessed various interventions to reduce TSE in children. We assessed the benefit of replicate objective measurements to assess exposure over a period of up to a month.

## METHODS

### Study selection

To identify datasets with repeated measures of child TSE that included both parental reports and objective measures, we searched the literature for original trials of interventions that attempted to reduce child exposure to tobacco smoke, involved participants who were the parent(s) (mother, father or both parents) of children aged 0–18 years; and in which the study outcome was the child’s TSE as reported by a parent and as measured using one or more biomarkers. We searched Web of Science, MEDLINE, BIOSIS Previews, Journal Citation Reports, and the Cochrane library, for articles published in English through the end of 2012. Search terms used with all databases are presented in Supplementary file S1.

Of 61 studies identified, most were longitudinal, with outcomes measured at several time points; some included replicate objective measurements at the same time point. We included studies with at least 3 longitudinal time points, at least 2 parental reports of their smoking behavior and/or child’s exposure at each time point, and sample sizes larger than 150. We were able to obtain raw data from 3 studies: Hovell et al.^18^ (2009), Kalkbrenner et al.^19^ (2010), and Wilson et al.^20^ (2011) (ClinicalTrials. gov Identifier: NCT00217958). We later included an additional study conducted by authors of this article, Rosen et al.^21^ (2021) (ClinicalTrials.gov Identifier: NCT02867241), although it assessed TSE at only two time points. These four studies will be referred to as the studies of Hovell, Kalkbrenner, Wilson, and Rosen, for brevity.

We explored the relationships between parent-reported and objective measures of child TSE from all children in the studies. We were particularly interested in understanding which, if any, of the parent-reported measures were most highly correlated with the objective measures. We were also interested in differences in correlations between measures, over time and by intervention arm. We assessed the evidence for a ‘learning curve’ whereby parents might report exposure more ‘accurately’ (that is, more consistently with the objective measures) over time, as a result of having been sensitized to the questions in the early trial period. Finally, in the two trials that used replicate objective measures, we examined within- and between-subject variation to understand whether replication yields information that provides a more useful exposure assessment than a single measure alone.

All the objective measures were analyzed after log transformations, as is common in the literature, due to their skewed distributions. We used data as provided to us by the authors of the studies.

### Statistical analyses

We performed the following analyses: associations between objective measures and parental report variables summarized by Pearson correlation coefficients. We used linear regression to discover which parent-reported measures best predicted the objective measure values obtained at the same time points. The regression models included time, group and their interaction and other potential explanatory variables: child-related, parent-related, sociodemographic and environmental variables (Supplementary file Tables S3–S10). We used forward selection to determine which parent-reported measures to include in the model and 10-fold cross-validation to determine which set of parent-reported measures best predicted the objective measure^22^.

We were interested in understanding if there was a learning curve over time, by which study participation led to increased parental awareness of TSE, and hence to parental responses more closely correlated with the objective measures. To assess trends in correlations over time, we regressed Fisher transformed^23^ correlation coefficients, $z^{\prime }=\frac{1}{2}\text{ ln }\left(\frac{1+r}{1-r}\right)$ against time.

To assess the value of replicate objective measures, we used restricted maximum likelihood to estimate within- and between-subject variation^18,20^.

In the analyses that included all time points, the longitudinal measures for each subject cause dependencies that render standard p-values inaccurate. For those analyses, we used bootstrap confidence intervals (CIs) for the correlations, with bootstrap samples taken at the subject level^24^.

The Wilson and the Hovell data included three baseline measurements of urinary cotinine on samples obtained at intervals of approximately 1–2 weeks (Wilson) and 1 week (Hovell). We used two analytic approaches, first computing the average of the three measurements followed by a log transformation, and secondly using each of the three baseline measurements independently. In subsequent analyses, the log of the averaged triplicate was used.

All studies asked multiple questions of the parent(s) about their and/or others’ smoking (overall, in the home, or in the car) and the child’s exposure to that smoking, but the sets of questions differed among the studies, and the specific wording differed in potentially important ways.

We use abbreviations for variable names (see the Abbreviations list above). For example, NCIGS (number of cigarettes) denotes heaviness of smoking among residents of the household. There were differences in the actual definition in different studies. The questions and definitions for each study are presented in Supplementary file Table S2. LSCot was defined as log serum cotinine, LUCot as log urinary cotinine, LHCot as log hair cotinine, LHNic as log hair nicotine, and LANic as log air nicotine.

## RESULTS

The four trials included 884 participants with children aged 0–12 years (Hovell: 150; Wilson: 352; Kalkbrenner: 223; Rosen: 159). The studies of Kalkbrenner and of Wilson, collected data at baseline, 6 and 12 months, Hovell at baseline, 3, 6, 12, and 18 months, and Rosen at baseline and 6–9 months later. Urinary cotinine was measured in the Hovell and the Wilson studies, hair nicotine in Rosen’s study and both hair cotinine and serum cotinine in the Kalkbrenner. Hovell and Kalkbrenner also measured home air nicotine (Table 1).

**Table 1:** Details of studies

	*Wilson et al.^20^ (2011)*	*Kalkbrenner et al.^19^ (2010)*	*Hovell et al.^18^ (2009)*	*Rosen et al.^21^ (2021)*
*Age of children (years)*	*3–12*	*Elementary-school-age 5–12*	*0–4*	*<8*
Time of data collection	Baseline, 6 and 12 months	Baseline, 6 and 12 months. Air nicotine was measured only at 6 and 12 months	Baseline, 3, 6, 12 and 18 months. Home air nicotine was collected from 50 randomly selected families at baseline and from 36 of the selected families at six months.	Baseline and 6–9 months
Objective data	Child urinary cotinine	Child serum cotinine, child hair cotinine and home air nicotine	Child urinary cotinine (from all children), and home air nicotine (from a sample of homes)	Hair nicotine
Sample size	352 (intervention 178, control 174)	223 (intervention 108, control 115)	150 (intervention 76, control 74)	159 (intervention 69, control 90)
Information at follow-up	Intervention 169, control 170, objective measure 341			155 parental reports, objective measure 146
Additional information			An additional objective in this study was to encourage parental smoking cessation. In both NCIGS* and NCIGSHOME**, one outlier was deleted from the analysis.	

* NCIGS: number of cigarettes.

** NCIGSHOME: number of cigarettes smoked in the home.

### Association between objective measures and parental reports

*Overview*

**Figure 1:**
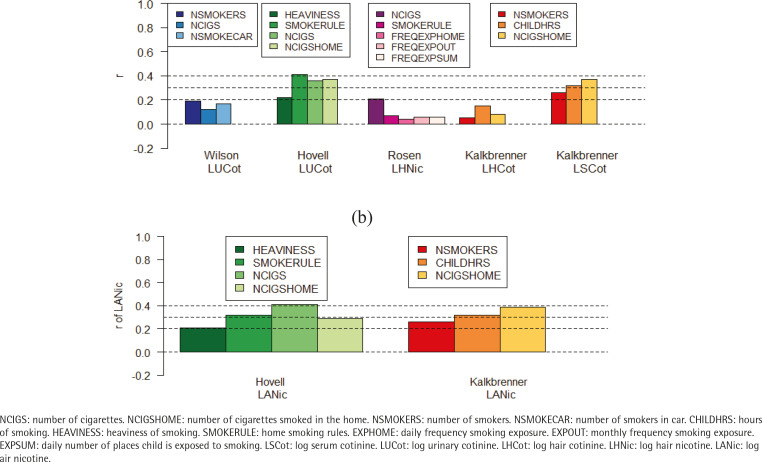
Correlations between objective measures and parent-reported measures across both intervention and control groups at all assessment points for each study. (a) Correlations between objective and parent-reported measures; (b) Correlations between objective environmental measure (LANic) and parent report variables, by study

**Figure 2:**
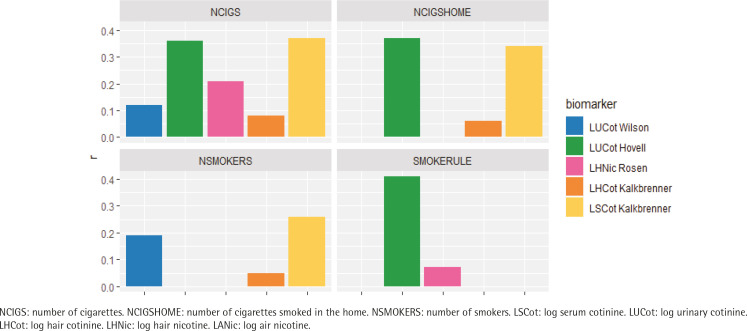
Comparing the correlations between objective measures and parent-reported measures across both intervention and control groups at all assessment points by parental measure of exposure

The correlation coefficients between objective measures and parent-reported measures ranged from moderate (highest: r=0.41) to essentially zero. Among the parent-reported measures, SMOKERULE had the highest correlations with the objective measure (r=0.41) followed by NCIGSHOME (0.39) or NCIGS (r=0.37). Objective measures LSCot and LUCot had better correlations with all the parent-reported measures than did the objective measures derived from hair (LSCot: range 0.26–0.37; LUCot: range 0.12–0.41; LHNic: range 0.04–0.21; LHCot: range 0.05–0.15) (Figures 1 and 2, and Table 2).

**Table 2:** Correlations between objective measures and parent-reported measures across both intervention and control groups at all assessment points for each study [r (p-value) n]

*Parental report*	*LUCot (Wilson)*	*LUCot (Hovell)*	*LHNic (Rosen)*	*LHCot(Kalkbrenner)*	*LSCot (Kalkbrenner)*	*LANic (Hovell)*	*LANic (Kalkbrenner)*
NCIGS	0.12 (0.003) 600	0.36 (<0.001) 604	0.21 (<0.0001) 287			0.41 (0.002) 77	
NCIGSHOME		0.37 (<0.001) 623		0.08 (0.066) 580	0.37 (<0.001) 627	0.29 (0.009) 80	0.39 (<0.0001) 397
NSMOKERS	0.19 (<0.001) 951			0.05 (0.25) 581	0.26 (<0.001) 628		0.26 (<0.0001) 398
NSMOKECAR	0.16 (<0.001) 636						
HEAVINESS		0.22 (<0.001) 612				0.21 (0.064) 82	
SMOKERULE		0.41 (<0.001) 642	0.07 (0.238) 268			0.32 (0.003) 86	
EXPHOME			0.06 (0.318) 285				
EXPOUT			0.04 (0.503) 284				
EXPSUM			0.06 (0.292) 283				
CHILDHRS				0.15 (<0.001) 581	0.32 (<0.001) 628		

NCIGS: number of cigarettes. NCIGSHOME: number of cigarettes smoked in the home. NSMOKERS: number of smokers. NSMOKECAR: number of smokers in car. CHILDHRS: hours of smoking. HEAVINESS: heaviness of smoking. SMOKERULE: home smoking rules. EXPHOME: daily frequency smoking exposure. EXPOUT: monthly frequency smoking exposure. EXPSUM: daily number of places child is exposed to smoking. LSCot: log serum cotinine. LUCot: log urinary cotinine. LHCot: log hair cotinine. LHNic: log hair nicotine. LANic: log air nicotine. For detailed descriptions of these questions, see Supplementary file Table S1.

*Effect of using a single baseline reading of LUCot versus the average of 3 readings*

Wilson and Hovell took multiple baseline measurements of urinary cotinine. In Wilson’s study similar correlations between urinary cotinine and parent-reported measures were observed when the average of the baseline cotinine measures was used as when the individual cotinine measures were used. In Hovell’s study, we found slightly, but not significantly, larger correlations between the average of the baseline cotinine measures and parent-reported measures than between the individual cotinine measures and the parent-reported measures. For example, the correlations of the 3 repeats of LUCot at baseline with SMOKERULE for the 3 separate measurements at baseline were r=0.42, 0.46, and 0.44, respectively. By contrast, after averaging the three measurements, the correlation was r=0.48 (Supplementary file Table S2).

*Prediction of objective measures by parental report*

Table 3 summarizes the cross-validation results according to study and objective measure. The first line for each objective measure gives the cross-validation mean square when ignoring all the parental report variables. Subsequent lines give the mean square when one or more parental report is used to predict the objective measure result. Comparison with the top line quantifies what fraction of the crossvalidated variation was explained by the regression model (Supplementary file Tables S3–S10).

**Table 3:** The regression analysis and cross-validation according to study and objective measure

*Study*	*Model^a^*	*MSE*	*R^2^*	*MSE*	*R^2^*	*MSE*	*R^2^*
**Kalkbrenner**	**Objective measure**	LSCot		LHCot		LANic	
	Variance	1.9		1.75		0.453	
	Intercept	1.91	0	1.75	0	0.454	0
	***Predictors based on one parental report***						
	NCIGSHOME (number of cigarettes smoked per day by all smokers in the home)	1.65	0.139	1.75	0.021	0.377	0.153
	CHILDHRS (hours of smoking in the same room as the child by everyone)	1.71	0.104	1.77	0.006	0.401	0.130
	NSMOKERS (number of smokers who live at home or spend time at home on a weekly basis)	1.78	0.069	1.78	0.002	0.42	0.068
	***Predictors with parental reports***						
	CHILDHRS + NCIGSHOME	1.58	0.177	1.75	0.022	0.36	0.194
	CHILDHRS + NSMOKERS	1.62	0.155	1.75	0.022	0.377	0.155
	NCIGSHOME + NSMOKERS	1.65	0.146	1.78	0.006	0.382	0.156
	CHILDHRS + NCIGSHOME + NSMOKERS	1.57	0.188	1.76	0.022	0.359	0.198
	***Predictors with parental reports and race***						
	NCIGSHOME + BLACK	1.52	0.206	1.23	0.313	0.375	0.161
	CHILDHRS + BLACK	1.65	0.142	1.24	0.310	0.403	0.104
	NSMOKERS + BLACK	1.70	0.117	1.25	0.297	0.421	0.069
	CHILDHRS + NCIGSHOME + NSMOKERS + BLACK	1.43	0.262	1.2	0.334	0.357	0.207
**Rosen**	**Objective measure**	LHNic					
	Variance	3.37					
	Intercept	3.4	0				
	***Predictors based on one parental report***						
	NCIGS (daily, both parents combined)	3.27	0.043				
	EXPSUM (exposure in the home, ranging from several times/day to never)	3.39	0.004				
	EXPOUT (exposure outside, ranging from several times/day to never)	3.35	0.002				
	***Predictors with parental reports***						
	NCIGS + EXPOUT	3.24	0.044				
	NCIGS + EXPSUM	3.26	0.044				
	NCIGS + EXPHOME	3.29	0.045				
	NCIGS + EXPOUT + EXPHOME	3.28	0.044				
	NCIGS + EXPOUT + EXPSUM	3.28	0.044				
	NCIGS + EXPHOME + EXPSUM	3.28	0.044				
	NCIGS + EXPOUT + EXPHOME + EXPSUM	3.28	0.044				
	NCIGS + SMOKERULE + EXPHOME + EXPSUM	3.36	0.040				
	NCIGS + EXPOUT + EXPHOME + SMOKERULE	3.36	0.040				
	NCIGS + EXPOUT + EXPHOME + EXPSUM + SMOKERULE	3.36	0.040				
**Hovell**	**Objective measure**	LUCot		LANic			
	Variance	1.24		14.4			
	Intercept	1.24	0	14.8	0		
	***Predictors based on one parental report***						
	SMOKERULE (how cigarette smoking is handled in the home: No one is allowed to smoke to smoking allowed anywhere in home (1-4)	1.05	0.165	13.4	0.104		
	NCIGSHOME (number of cigarettes smoked per week by all smokers in the home)	1.1	0.134	15.4	0.084		
	NCIGS (total number of cigarettes per week that were smoked by all users at home and at all other places the child was present during the week)	1.12	0.131	15.0	0.168		
	***Predictors with parental reports***						
	HEAVINESS + SMOKERULE	0.99	0.173	14.4	0.115		
	NCIGSHOME + SMOKERULE	1.02	0.199	14.9	0.127		
	NCIGS + SMOKERULE	1.02	0.209	14.8	0.206		
	NCIGS + HEAVINESS + SMOKERULE	0.973	0.211	15.6	0.199		
	NCIGS + NCIGSHOME + SMOKERULE	1.03	0.213	14.1	0.268		
	NCIGS + NCIGSHOME + HEAVINESS	1.04	0.159	15.6	0.222		
	NCIGS + NCIGSHOME + HEAVINESS + SMOKERULE	0.977	0.216	15.1	0.260		
	***Predictors with parental reports and race***			15.9	0.083		
	SMOKERULE + BLACK	1.05	0.166	13.5	0.112		
	NCIGSHOME + BLACK	1.10	0.138	15.5	0.084		
	NCIGS + BLACK	1.12	0.134	15	0.169		
**Wilson**	**Objective measure**	LUCot					
	Variance	3.07					
	Intercept	3.07	0				
	***Predictors based on one parental report***						
	NSMOKECAR (number of smokers in car)	2.78	0.028				
	NCIGS (total number of cigarettes per day by all users)	2.79	0.014				
	NSMOKERS (number of smokers)	2.94	0.036				
	***Predictors with parental reports***						
	NCIGS + NSMOKECAR	2.76	0.029				
	NCIGS + NSMOKERS + NSMOKECAR	2.77	0.032				
	NCIGS + NSMOKERS	2.78	0.021				
	***Predictors with parental reports and race***						
	NCIGS + NSMOKECAR + BLACK	2.66	0.068				
	NCIGS + NSMOKERS + NSMOKECAR + BLACK	2.67	0.070				
	NCIGS + BLACK	2.68	0.055				
	NSMOKECAR + BLACK	2.68	0.064				

a We put in the table only the 3 best models for each study and biomarker.

NCIGS: number of cigarettes. NCIGSHOME: number of cigarettes smoked in the home. NSMOKERS: number of smokers. NSMOKECAR: number of smokers in car. CHILDHRS: hours of smoking. HEAVINESS: heaviness of smoking. SMOKERULE: home smoking rules. EXPHOME: daily frequency smoking exposure. EXPOUT: monthly frequency smoking exposure. EXPSUM: daily number of places child is exposed to smoking. LSCot: log serum cotinine. LUCot: log urinary cotinine. LHCot: log hair cotinine. LHNic: log hair nicotine. LANic: log air nicotine.

When using LSCot, NCIGSHOME best predicted the objective measure (R^2^=0.139). When using LUCot, SMOKERULE and NCIGSHOME or NCIGS gave the best predictions (R^2^=0.165, 0.134, 0.131, respectively). LHCot and LHNic had weak correlations with the parental reports (max R^2^=0.021 and 0.043, respectively) and were not accurately predicted by any combination of them. The accuracy in prediction of objective measures from parental reports was stable across time and group, as evidenced by their similar mean square errors (MSE).

The strongest predictive relationship was found for urinary cotinine in Hovell’s study and included the variables home smoking rules and total number of cigarettes smoked in the home (R^2^=0.209). Additional explanatory variables found to be significantly related to the objective measures in more than one study were: gender (female gender was associated with greater exposure), number of rooms in the house (more rooms were associated with lower exposure), assessment time point (later assessment points were associated with lower levels), race (the objective measure was higher in Black than in White participants) and income (negative association).

### Analysis of change in correlations over time

We did not find any consistent time trend in correlations between parental report variables and objective measures in the different studies, and time was generally not a statistically significant predictor. For details see Supplementary file Table S2.

### Assessing the value of replicate measures

Supplementary file Table S11 shows the estimated within- and between-subject variances in the Hovell and Wilson studies for multiple baseline measurements taken within a reasonably short span of time with no intervening intervention (e.g. three measurements of LUCot at baseline in both Hovell and Wilson). The between-subject variances were similar (0.65 and 0.57, respectively). However, the within-subject variances differed substantially (0.22 and 3.15, respectively).

## DISCUSSION

The four exposure-reduction intervention trials considered in this analysis show that researchers in the field of child TSE use different objective measures (urinary cotinine, serum cotinine, hair cotinine, hair nicotine), different time periods for measurement of air nicotine (1 week, 6 months), and different sets of parental questions about smoking in the home and about the child’s TSE. Differences in wording likely affected how parents responded and hence the correlation of parental reports with objective measures. The studies also differed in the number of assessments and their spacing.

The correlations between objective measures and parent reports in these studies ranged from moderate (r=0.41) to non-existent (r=0). Objective measures most closely related to parental reports were serum cotinine and urinary cotinine, whereas hair nicotine and hair cotinine had the lowest associations. Parental questions most closely related to the objective measures were the number of cigarettes smoked by parents and/or others, and home smoking rules.

The best predictive model we found was from the study of Hovell, relating urinary cotinine to home smoking rules and total number of cigarettes smoked in the home. Additional explanatory variables were significantly related to the objective measures in more than one study but were not recorded in all the studies.

### Regarding correlations between objective measures and parental report

Poor synchronization between the time period measured by a given biomarker, and the time period assessed by parental reports, could partially explain low correlations. Our observations regarding this issue differed across studies. For example, Kalkbrenner used serum cotinine (24–48 hours exposure) and hair cotinine (several months exposure). Their questionnaire referred to the child’s exposure in the past 24 hours, suggesting, *a priori*, that there should be a higher correlation with serum cotinine than with hair cotinine. Indeed, this was borne out empirically (r=0.37, p<0.001 with LSCot; and r=0.08, p=0.066 with LHCot). By contrast, the study of Rosen found that hair nicotine had higher correlations with exposure during the previous day (r=0.21) than in the recent month (0.04–0.06).

Biomarker expression may differ across individuals. For example, hair nicotine has been found to be associated with hair color^25^.

It may be that parental reporting of the number of cigarettes smoked is unreliable and/or systematically biased toward over-/under-reporting. However, even if parents are reporting reliably, the questions may not be sufficiently detailed to capture the full range of possible situations and so assess true exposure. For example, assuming that a reasonably strong association exists between the number of cigarettes smoked in a home and child exposure, the observed correlation may be reduced because individual children spend varying amounts of time indoors – and perhaps less so as they mature. Home architecture may differ, ventilation may differ, and parents may smoke in different areas or in different amounts. This complexity demonstrates the importance of valid biomarkers for exposure measurement and the importance of developing questions which better capture exposure.

Similar modest correlations were found in the literature. Tang et al.^12^, in their summary of the literature, reported correlations of 0.22–0.69 between hair nicotine and parental report (see also Supplementary file Table S5), -0.02–0.77 for urinary cotinine (see also Supplementary file Table S6), and 0.34–0.35 for serum cotinine (see also Supplementary file Table S7). Modest correlation was found between number of cigarettes smoked per day and air nicotine r=0.36, and r=0.44 for urinary cotinine^26^, and lower correlation between number of cigarettes smoked by the father and hair nicotine from the father, their spouse and the child (r=0.13; r=0.07, and r=0.03, respectively)^15^. Similar results were found in infants, with correlations from r=0.35 with plasma nicotine and number of days the infant was exposed to TSE, to a maximum of r=0.66 between hair nicotine and number of cigarettes smoked per day^27^. Another recent study found a large range of correlations, from -0.2 to +0.4, which were not statistically significant, between parent-reported measures and air and hair nicotine^28^. However, one study found higher and statistically significant correlations between maternal hair nicotine and parent-reported measures (r=0.46–0.74)^29^.

Other fields also suffer from low correlations between objective measures and reported exposure. For instance, in nutritional epidemiology, most correlations that Trabulsi and Schoeller^30^ found between dietary energy intake and doubly labeled water were not statistically significant and ranged from r=0.06 to r=0.86. Other studies also reported low correlations: Freedman et al.^31^ found that the correlations between reported (via three 24-hour recall food frequency questionnaires) and measured energy intake ranged from r=0.21 to r=0.49. Among females, the correlations between data from food recall questionnaires and measured sodium:potassium ratio ranged from r=0.15 to r=0.64^32^.

Our analysis found that urinary and serum cotinine had higher correlations with parental report than did hair nicotine. Our results differ from those of Al-Delaimy et al.^33^, who found parental report to be more highly correlated with hair nicotine than with urinary cotinine, leading them to conclude that hair nicotine was a ‘more precise biomarker of exposure than urinary cotinine’.

### Learning curve

Prior to performing the analyses, we had hypothesized that there might be a ‘learning curve’ in which parents would report child exposure more accurately over the course of a study. In principle, more accurate reporting should lead to higher correlations at later times. No evidence of increased correlations between reported and observed measures over the course of the study was found in any of the four studies when the data from all groups were examined together. When examined separately, the results were inconsistent. Our conclusion does not support the hypothesis that correlations increase over time.

### Regarding replicate samples of biomarkers

Exposure of a child may vary significantly from one day to the next. Replicates across a short time window enable us to assess the variation and to provide a more precise assessment of typical exposure^34^. As with any measure, results will also be affected by other sources of measurement variation such as sampling techniques and laboratory precision, including limits of detection and quantification^35,36^.

Many factors need to be considered in deciding whether to take short-term replicates. Recruitment, screening, and assessment of additional subjects is costly, especially in trials that examine individually delivered behavioral interventions. These must be weighed against the cost for collection and analysis of replicate samples for each subject. Further, relative costs may vary between countries and research settings.

An important consideration in intervention trials is to maximize power for finding effects on outcome. Greater power may be achieved by maximizing sample size, while taking a single objective measure from each participant at baseline and at the end of the study, or by reducing within-subject variance in the exposure outcome measure. Careful evaluation is needed to assess the trade-off between the expense of the replications and the expense of recruitment and trial participation, to achieve the desired power for study comparisons. Bitan et al.^37^ provided a design framework for studies with both objective measures and self-reports and found that excessive replication typically compromises power.

We think that decisions on replication should relate to the relative sizes of the inter-participant and intraparticipant variation. It is noteworthy that we found a large difference in intra-participant variation between the Hovell and Wilson studies, although both assessed the same biomarker. This could reflect differences between study populations. Hovell included healthy children from poor families in San Diego California whose mothers reported smoking ≥10 cigarettes per week, whereas Wilson included asthmatic children from the San Francisco Bay area who had minimum urinary cotinine of 10 ng/mL and no socioeconomic targeting. The large variance in Wilson’s study makes replicates more attractive, but this differs from Hovell’s study, in which the intra-subject variation was considerably smaller. Good estimates of these components are required at the study design stage.

We disagree with the recommendation of Matt et al.^38^ that one should take enough replicate samples so that the within-subject averages have a variance that is roughly 1% of the total variance. This reasoning led them to recommend as many as 9–13 replicates of urinary cotinine. We are convinced that this criterion sets much too high a bar for measurement precision at the individual level and, if followed, would lead to wasted resources and an unjustified burden on research subjects.

### Strengths and limitations

A major strength of this research was its analysis of raw data from multiple studies. Our research included four non-randomly chosen intervention trials. Each of these trials is one example of how researchers currently approach the issue of child TSE assessment in field conditions (as opposed to in a laboratory setting, where a validation study might be conducted). Although the studies were all published prior to 2012, they include all the primary biomarkers currently in use for measuring passive exposure to tobacco smoke. A limitation is the small number of included studies and the fact that the questions presented to parents were not consistently asked in every study, and even reasonably parallel questions had variations in wording that would plausibly affect parental reporting. This may well be reflective of the variation in research practices in this field, given the lack of standardization of questions. Also, some variables that were available in the various datasets, but not presented in the previous publications, were not analyzed. It is possible that since these studies were conducted, there have been improvements in analytical methods, reducing lower limits of detection and providing more accurate biomarker measurement. If so, recent studies of biomarkers may provide more accurate measurements of exposure. These four studies cannot be considered to be representative of all research in the field, and the fact that no single trial included all possible objective measures and questions means we are unable to make definitive statements about advantages of different querying methods.

### Implications for further research

*Biomarkers*

- It is well-accepted that biomarkers are preferred for assessing exposure. Based on the correlations found in these studies, exposure assessments should include biomarkers. A better understanding of the sources of variation associated with objective measures is important. This will help ensure that the full extent of exposure is measured and that demographic factors that affect the results can be taken into account when interpreting the results.
- Multiple measurements are required to assess long-term exposure trends. Multiple measurements within a relatively short time span contribute to more precise assessment for each individual participant, including comparison of pre- and post-study exposure. The trade-off in resource requirements between increasing the number of participants and taking multiple measurements on each participant requires a careful assessment of study goals, the costs of patient recruitment and intervention delivery, as well as the costs of data collection. Bitan et al.^37^ provide a valuable assessment framework to achieve adequate statistical power in comparisons between an intervention group and a control group.

*Parental reports*

- Parent-reported data should be collected, as they provide useful, complementary information. First, the ability to contrast parental reports with biomarkers can act as a check against possible gross errors in objective measures, and also may identify parental misperceptions of a child’s exposure^39^, which may assist in encouraging the parent to reconsider their subjective assessment. Second, in an intervention trial, it can provide detailed information on the sources of TSE that may be helpful in guiding parents in the intervention group to reduce exposure. Third, parental report measures may be useful in assessing secondary outcomes of the trial which are related to behavioral changes, such as changes in smoking behavior.
- It is imperative to standardize a set of questions that adequately describe all sources of child exposure, especially in the home and the wider home environment (porches, hallways, gardens, stairwells), as well as in cars, childcare, and other environments; from primary caretakers, residents, grandparents, educators, and visitors. In addition to combustible cigarettes, questions should ensure that exposure to pipes, cigars, narghile, electronic cigarettes and heated tobacco products, as well as secondhand smoke exposure, is included. The importance of including various possible sources is likely to vary across different cultural groups and local regulations on smoking in public areas.

*Correlations*

- In the context of the four trials included in this study, we found that the parent-reported measures most highly correlated with objective measures were: 1) number of cigarettes smoked, and 2) the nature of rules regarding smoking in the home. When used together, the predictive value was still low, but was greater than for either one separately. This suggests that it may be useful to include both questions when asking parents about their child’s exposure.

The low correlations between biomarkers and parental reports may imply that these measures reflect different aspects of exposure. Methods have recently been developed to produce improved measures of exposure that combine the information from objective measurements and self-reports^40^. This is a promising direction which needs to be investigated further. Another benefit of further research is to assess the impact of improvement in analytical methods.

Further meta-analysis of more recent studies that involved both objective measurement of exposure and parent-reported exposure also is needed, focusing particularly on studies that used comparable questions to elicit parent report. Such eta-analysis might also include observational studies (cross-sectional or longitudinal), as well as intervention trials.

## CONCLUSIONS

Correlations between objective and parent-reported measures of child TSE were modest at best in the four RCTs included in this study. Biomarkers most closely related to parental report were serum cotinine and urinary cotinine. Parental questions most closely related to biomarkers were number of cigarettes and home smoking rules; together these formed the best predictive question set. Within-subject repeatability of biomarker measures varied across studies, suggesting that direct pilot data are needed to assess the benefit of replicate measurements.

Improvements in objective and parent-reported child exposure measurement are needed to accurately estimate children’s TSE.

## Data Availability

The data supporting this research are available from the authors on reasonable request.
