# Supplementary material for: Association between objective measures and parent-reported measures of child tobacco smoke exposure: A secondary data analysis of four trials
Source: Tob Induc Dis. 2022 Jun 29;20:62. doi: 10.18332/tid/150296 (PMC9241496; doi:10.18332/tid/150296)
Supplement: Supplementary file 1 [file TID-20-62-s1.pdf]

Supplementary Material

Supplementary S1: search terms. Supplementary Table S1: Parental report questions and objective measurement by study

Supplementary Table S2: Correlations with log urinary cotinine at baseline

Supplementary Table S3: Results from the linear regression of log urinary cotinine in Wilson's study with the parental reports and all demographic variables as explanatory variables

Supplementary Table S4: Results from the linear regression of log urinary cotinine in Hovell's study with the parental reports and all demographic variables as explanatory variables

Supplementary Table S5: Results from the linear regression of log hair nicotine in Rosen's study the parental reports and all demographic variables as explanatory variables

Supplementary Table S6: Results from the linear regression of log hair cotinine in Kalkbrenner's study with the parental reports and all demographic variables as explanatory variables

Supplementary Table S7: Results from the linear regression of log serum cotinine in Kalkbrenner's study with the parental reports and all demographic variables as explanatory variables

Supplementary Table S8: Results from the linear regression of log air nicotine in Hovell's study with the parental reports and all demographic variables as explanatory variables

Supplementary Table S9: Results from the linear regression of log air nicotine in Rosen's study with the parental reports and all demographic variables as explanatory variables

Supplementary Table S10: Results from the linear regression of log air nicotine in Kalkbrenner's study with the parental reports and all demographic variables as explanatory variables

Supplementary Table S11: Estimates of within and between subject variance in Hovell and Wilson's studies

Supplementary Figure S1: scatter plots for the most highly correlated measurement in Hovell's study

Supplementary S2: additional analysis on Wilson's study**.**

Supplementary S1: Search terms: children Passive Exposure to Tobacco Smoke Saliva/urine cotinine/hair cotinine, child exposure to SHS parental report and objective measure/urine cotinine/hair cotinine/saliva cotinine, child exposure to SHS questionnaire and urine cotinine/hair cotinine/saliva cotinine, child exposure to SHS questionnaire and nicotine/cotinine, child exposure to tobacco smoke exposure questionnaire and cotinine/nicotine, child exposure to tobacco smoke exposure parental report and cotinine/nicotine.

Supplementary Table S1: Parental report questions and objective measurement by studyIn reporting the results, we use abbreviations for variable names. For example, NCIGS (number of cigarettes) denotes heaviness of smoking among residents of the household. There were differences in the actual definition in different studies. In the Rosen study, it meant total number of cigarettes smoked by both parents per day. In the Hovell study, it meant total number of cigarettes per week that were smoked by all users at home and at all other places the child was present during the week. NCIGSHOME was defined by Kalkbrenner and Hovell as number of cigarettes smoked per day\week by all smokers in the home. SMOKERULE was the variable used to assess home smoking rules or practices, i.e., whether cigarette smoking was allowed in the home, restricted in some manner inside the home, or not allowed in the home, as defined in Hovell's and Wilson's studies, or where parents usually smoked when they did so, in Rosen's study. For the objective measures, LSCot was defined as log serum cotinine, LUCot as log urinary cotinine, LHCot as log hair cotinine, LHNic as log hair nicotine and LANic as log air nicotine.

Topic and variable name in present study	Wilson	Kalkbrenner	Hovell	Rosen	Name used
Parental report questions					
Number of cigarettes	total number of cigarettes per day by all users: this variable summarizes several different questions, which relate to the number of cigarettes smoked by each smoker living in the house		number of cigarettes per week smoking in the same room (or car) as the child from all sources and all locations: this variable summarizes several different questions, which relate to how many cigarettes were smoked in the past 7 days by anyone: in the home, car, a relative's house, a friend's house, babysitter's/ daycare, your workplace, social event, sport event, public place, any other place	total number of cigarettes smoked by both parents per day: How many cigarettes does the mother of the child smoke everyday and how many does the father smoke?	NCIGS

Number of cigarettes smoked in the home		number of cigarettes per day smoked in the home by all smokers (cig/day): this variable summarizes several different questions, which relate to About how many cigarettes per day does the following people smoke at home: mother, father, siblings, grandparents, other individuals who live in the home, other individuals who spends time at home on a weekly basis smoke	number of cigarettes per week smoked in the home by all smokers: how many cigarettes does anyone who lives in your home or visited your home in the past 7 days smoke inside your home during the past 7 days		NCIGSHOME

Number of smokers	number of smokers: this variable summarizes several different questions, which relate to smoking status of all people living in the house	number of smokers who live at home or spend time at home on a weekly basis. Do any of the following smoke cigarettes: mother/father/siblings/grand parents/other individuals who live in the home/other individuals who spend time at home on a weekly basis.			NSMOKERS
Number of smokers in car	number of smokers in car: this variable summarizes several different questions, which relate to the questions does s/he smoke in the car when child is riding in it				NSMOKECAR

Hours of smoking		hours of smoking in the same room as the child by everyone: About how many hours yesterday was there smoking in the same room as the child from anyone in your home?			CHILDHRS
Heaviness of smoking			parent's level of smoking (0-6)		HEAVINESS
Home smoking rules			how is cigarette smoking handled in the home: No one is allowed to smoke in Home/Only special guests allowed / Only allowed in certain areas / Smoking allowed anywhere in home (1-4)	where parents usually smoked when they did so: whole house/inside at a window or on an indoor balcony/on an outdoor balcony/away from the home (1-4)	SMOKERULE

Daily frequency smoking exposure				Frequency exposure at home in the last month	EXPHOME
Monthly frequency smoking exposure				Frequency exposure outside the home in the past month	EXPOUT
Daily number of places child is exposed to smoking				Frequency of exposure in all places in the past month	EXPSUM
Biomarakers and objective measurements					
Log serum cotinine		LU			LSCot
Log urinary cotinine	Log urinary cotinine		Log urinary cotinine		LUCot
Log hair cotinine		Log hair cotinine			LHCot
Log hair nicotine				Log hair nicotine	LHNic
Log air nicotine		Log air nicotine	Log air	nicotine	LANic

Table S2: correlations with log urinary cotinine at baseline

Study	Self reported measure	Averaging the three measurement	First measurement	Second measurement	Third measurement
Wilson	NSMOKERS	0.01	-0.03	0.05	0.02
	NCIGS	0.05	-0.02	-0.01	0.12
	NSMOKECAR	0.01	0.05	-0.08	0.04
Hovell	NCIGS	0.48	0.4	0.43	0.46
	NCIGSHOME	0.51	0.44	0.44	0.52
	SMOKERULE	0.48	0.42	0.46	0.44
	HEAVINESS	0.25	0.21	0.23	0.23

Table S3: Results from the linear regression of log urinary cotinine in Wilson's study with the parental reports and all demographic variables as explanatory variables

Param	B	SE	t	p.v
(Intercept)	3.027	0.335	9.039	0.000
NCIGS	0.024	0.005	4.564	0.000
Education	0.024	0.104	0.233	0.816
Married vs. Not Married	-0.048	0.155	-0.311	0.756
Income	-0.372	0.113	-3.299	0.001
Gender male vs. female	0.357	0.135	2.655	0.008
Age of child	0.001	0.022	0.028	0.977
Rooms	-0.072	0.031	-2.304	0.022
Ethnicity black vs. white	0.566	0.153	3.690	0.000
Time	-0.129	0.019	-6.682	0.000
Group control vs. intervention	-0.008	0.193	-0.042	0.967
time:group control vs. intervention	-0.017	0.026	-0.658	0.511

Table S4: Results from the linear regression of log urinary cotinine in Hovell's study with the parental reports and all demographic variables as explanatory variables

**Param**	**β**	**SE**	**t**	**p.v**
(Intercept)	30.722	12.012	2.558	0.011
NCIGS	0.003	0.001	5.165	0.000
SMOKERULE	0.246	0.039	6.367	0.000
Gender female vs. male	-0.130	0.084	-1.547	0.122
Ethnicity black vs. white	0.167	0.140	1.188	0.235
Mother's education	-0.029	0.019	-1.557	0.120
Child Age	-0.077	0.036	-2.154	0.032
Mother's Age	-0.014	0.006	-2.336	0.020
Number of rooms in the house	-0.054	0.022	-2.489	0.013
Time	-0.016	0.009	-1.817	0.070
Group control vs. intervention	-0.283	0.125	-2.259	0.024
time:group control vs. intervention	0.029	0.013	2.306	0.021

Table S5: Results from the linear regression of log hair nicotine in Rosen's study the parental reports and all demographic variables as explanatory variables

Param	β	SE	t	p.v
(Intercept)	0.658	1.168	0.564	0.573
NCIGS	0.029	0.013	2.216	0.028
SMOKERULE	0.082	0.166	0.492	0.623
EXPHOME	-0.136	0.068	-1.999	0.047
EXPOUT	-0.016	0.076	-0.213	0.832
Child gender male vs. female	-0.158	0.261	-0.604	0.547
Maternal education above high school vs. high school	-0.613	0.441	-1.389	0.166
Maternal education academic degree vs. high school	-0.210	0.358	-0.587	0.558
Paternal education above high school vs. high school	0.050	0.351	0.143	0.887
Paternal education academic degree vs. high school	-0.250	0.320	-0.780	0.436
Child age	-0.004	0.006	-0.619	0.536
Religiosity religious vs. Haredi	0.137	0.812	0.168	0.867
Religiosity traditional vs. Haredi	0.056	0.663	0.084	0.933
Religiosity secular vs. Haredi	-0.208	0.654	-0.318	0.750
Religiosity other vs. Haredi	-2.811	1.977	-1.422	0.156
Number of children at home	-0.005	0.126	-0.037	0.971
SES above vs. average and below	-0.089	0.275	-0.322	0.748
Maternal work	0.048	0.258	0.185	0.854
Paternal work	-0.255	0.319	-0.800	0.425
Family status parents live together vs. not live together	-1.498	0.662	-2.261	0.025
Group Control vs. intervention	-0.103	0.330	-0.312	0.755

time	-0.179	0.059	-3.010	0.003
time:group control vs. intervention	0.012	0.078	0.152	0.879

Table S6: Results from the linear regression of log hair cotinine in Kalkbrenner's study with the parental reports and all demographic variables as explanatory variables

Param	β	SE	t	p.v
(Intercept)	-1.158	0.426	-2.720	0.007
NSMOKERS	0.321	0.076	4.233	0.000
CHILDHRS	0.072	0.020	3.580	0.000
Married vs. not married	-0.024	0.117	-0.207	0.836
Mom's education	0.016	0.051	0.311	0.756
Ethnicity black vs. white	1.163	0.105	11.024	0.000
IncomeMidPoint	-0.064	0.000	-2.319	0.021
Gender male vs. female	0.016	0.093	0.168	0.867
Age of child	-0.001	0.026	-0.044	0.965
HOME	-0.030	0.007	-4.258	0.000
Season Spring vs. summer	-0.316	0.124	-2.554	0.011
Season Winter vs. summer	-0.407	0.123	-3.313	0.001
Season fall vs. summer	-0.325	0.129	-2.522	0.012
Home_vol	-0.119	0.001	-2.305	0.022
Time	0.012	0.013	0.914	0.361
Group control vs. intervention	0.055	0.141	0.388	0.698
time:group control vs. intervention	0.007	0.018	0.369	0.713

Table S7: Results from the linear regression of log serum cotinine in Kalkbrenner's study with the parental reports and all demographic variables as explanatory variables

Param	β	SE	t	p.v
(Intercept)	0.379	0.427	0.888	0.375
NSMOKERS	0.414	0.087	4.737	0.000
NCIGSHOME	0.027	0.005	5.135	0.000
CHILDHRS	0.112	0.022	5.140	0.000
Married vs. not married	-0.024	0.118	-0.202	0.840
Mom's education	0.090	0.051	1.767	0.078
Ethnicity black vs. white	0.265	0.109	2.427	0.016
IncomeMidPoint	-0.146	0.000	-5.265	0.000
Gender male vs. female	-0.441	0.093	-4.743	0.000
Age of child	0.037	0.025	1.445	0.149
HOME	-0.017	0.007	-2.458	0.014
Season Spring vs. summer	0.276	0.122	2.265	0.024
Season Winter vs. summer	0.236	0.121	1.945	0.052
Season fall vs. summer	0.127	0.126	1.007	0.315
Home_vol	-0.348	0.001	-6.605	0.000
Time	0.007	0.013	0.561	0.575
Group control vs. intervention	-0.007	0.141	-0.051	0.960
time:group control vs. intervention	-0.003	0.018	-0.181	0.857

Table S8: Results from the linear regression of log air nicotine in Hovell's study with the parental reports and all demographic variables as explanatory variables

**Param**	**β**	**SE**	**t**	**p.v**
(Intercept)	57.868	158.341	0.365	0.716
SMOKERULE	0.960	0.415	2.315	0.024
Gender female vs. male	-0.880	1.026	-0.858	0.395
Ethnicity black vs. white	-1.404	2.454	-0.572	0.569
Mother's education	-0.017	0.259	-0.065	0.949
Child Age	-0.268	0.407	-0.659	0.512
Mother's Age	-0.027	0.080	-0.339	0.736
Number of rooms in the house	-0.310	0.276	-1.124	0.266
Time	-0.483	0.218	-2.213	0.031
Group control vs. intervention	-2.223	1.248	-1.782	0.080
time:group control vs. intervention	0.712	0.324	2.197	0.032

Table S9: Results from the linear regression of log air nicotine in Rosen's study with the parental reports and all demographic variables as explanatory variables

Param	β	SE	t	p.v
(Intercept)	-1.430	2.069	-0.692	0.494
SMOKERULE	0.879	0.380	2.310	0.027
Gender female vs. male	-1.070	0.588	-1.820	0.078
Mother's education	-0.205	0.313	-0.654	0.518
Child age	0.039	0.136	0.288	0.775
Religion Christian vs. Jews	2.457	1.851	1.328	0.193
Religiosity	-0.099	0.216	-0.460	0.649
Number of kids at home	0.533	0.257	2.075	0.046
SES	0.005	0.213	0.022	0.983
Mom's work partial vs. full time	-0.242	0.771	-0.314	0.756
Mom's work not wokring vs. full time	0.078	0.717	0.109	0.914
Father's education	-0.684	0.269	-2.542	0.016
Time	0.090	0.073	1.239	0.224

Table S10: Results from the linear regression of log air nicotine in Kalkbrenner's study with the parental reports and all demographic variables as explanatory variables

Param	β	SE	t	p.v
(Intercept)	0.609	0.299	2.034	0.043
NCIGSHOME	0.019	0.003	6.484	0.000
CHILDHRS	0.057	0.014	4.006	0.000
Married vs. not married	-0.001	0.076	-0.019	0.985
Mom's education	-0.013	0.033	-0.401	0.689
Ethnicity black vs. white	-0.019	0.072	-0.265	0.791
IncomeMidPoint	-0.029	0.000	-1.610	0.108
Gender male vs. female	-0.164	0.061	-2.673	0.008
Age of child	0.021	0.017	1.283	0.200
HOME	0.004	0.005	0.795	0.427
Season Spring vs. summer	0.250	0.082	3.036	0.003
Season Winter vs. summer	0.229	0.081	2.831	0.005
Season fall vs. summer	0.202	0.084	2.408	0.017
Home_vol	-0.18	0.000	-5.211	0.000
Time	-0.010	0.014	-0.733	0.464
Group control vs. intervention	-0.286	0.189	-1.514	0.131
time:group control vs. intervention	0.030	0.020	1.514	0.131

Table S11: Estimates of within and between subject variance in Hovell and Wilson's studies

	**Between**	**Within**
Hovell	0.65	0.22
Wilson	0.57	3.15

Figure S1: scatter plots for the most highly correlated measurement in Hovell's study

Figure 1a: In Hovell's data scatter plot for log urinary cotinine and number of cigarettes smoked at home

Figure 1b: In Hovell's data scatter plot for log urinary cotinine and home smoking rules

Figure 1c: In Kalkbrenner's data scatter plot for log serum cotinine and number of cigarettes smoked at home

Figure 1a:

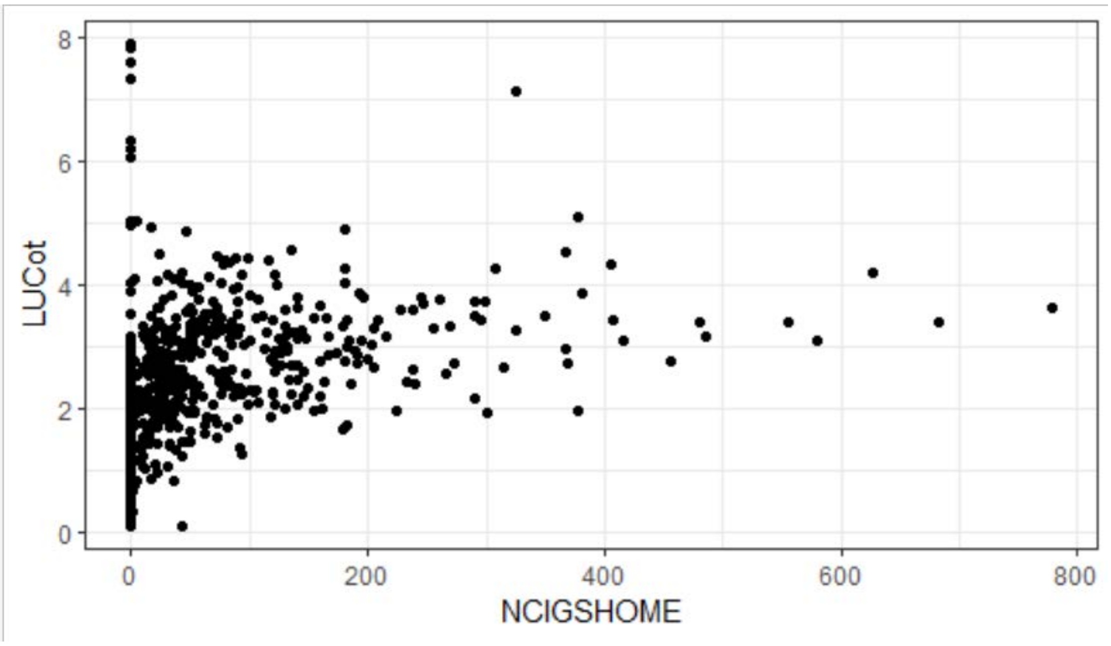

Figure 1b:

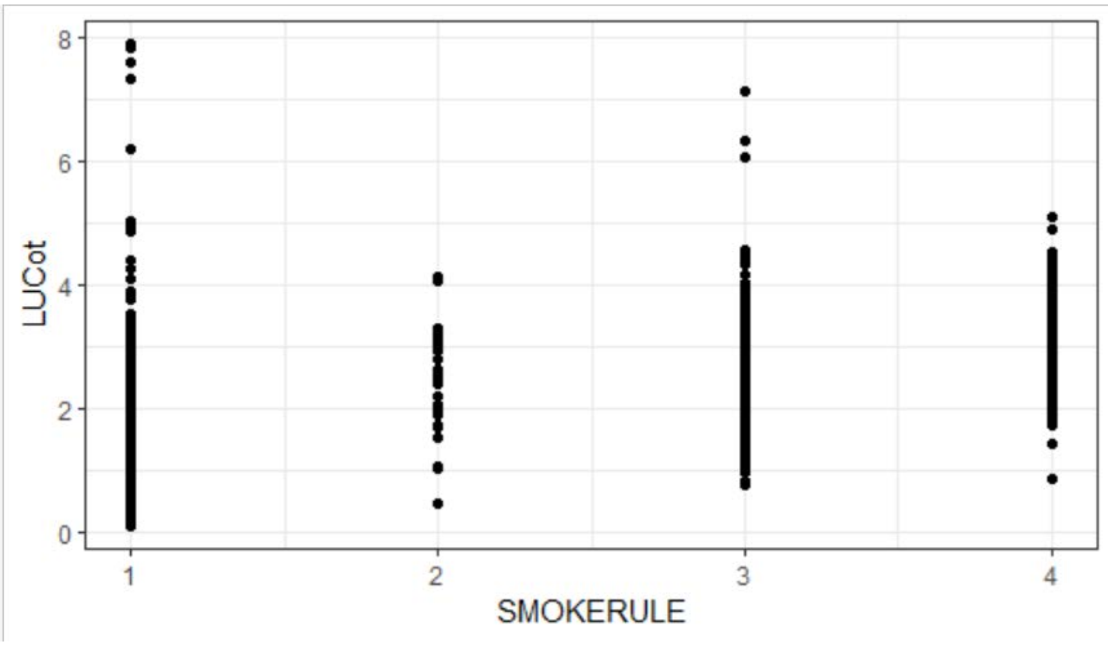

Figure 1c:

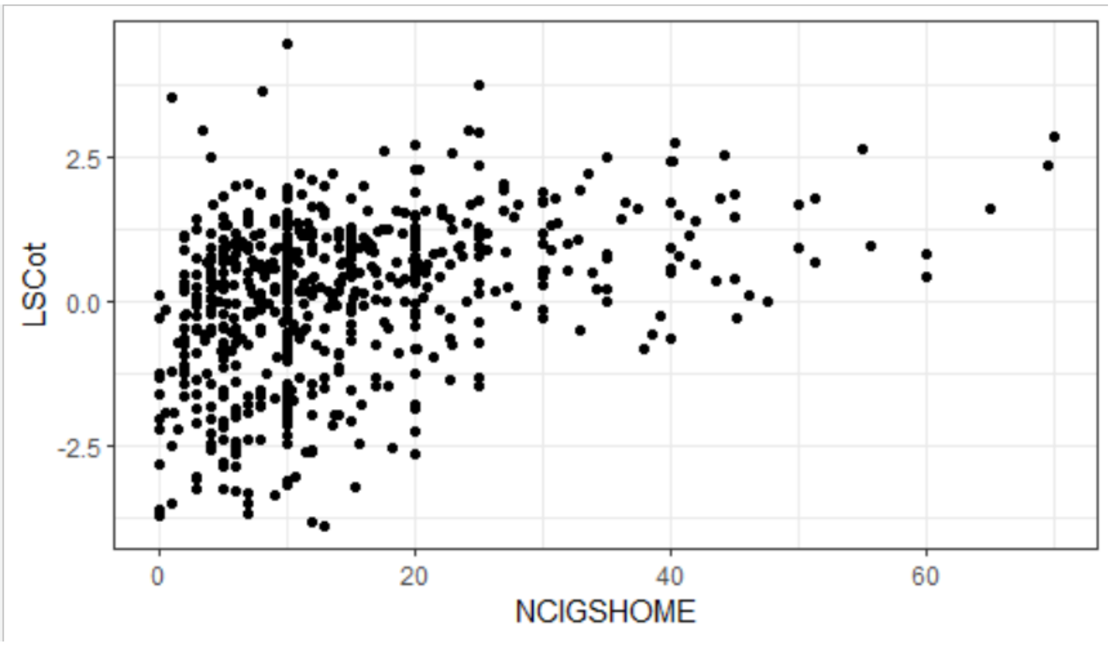

Supplementary S2:

In Wilson's study, there tended to be an increase in the correlations over time, although only one was statistically significant: the correlations between NSMOKERS and LUCot increased with time (baseline r=0.01, 6 months and 12 months r=0.21, p =0.0096). In Hovell's study, a statistically significant temporal trend (generally, lower correlations at later assessments) was found for the correlations between LUCot and NCIGS (baseline r=0.48, 3 months r=0.44, 6 months r=0.36, 12 months r=0.24, 18 months r=0.35, p=0.0487), and NCISHOME (baseline r=0.52, 3 months r=0.42, 6 months r=0.34, 12 months r=0.24, 18 months r=0.37, p=0.0346). There was also a statistically significant temporal trend (reduction) in the correlation between NCIGSHOME and LANic (baseline r=0.49, 6 months r=0.06, p =0.0425). In Rosen's study, there was no statistically significant change in the correlations between LHNic and any of the parental measures, and in Kalkbrenner's study, as well, there were no statistically significant trends in the correlations between LHCot and LSCot and any of the parental measures.

In both Wilson's and Hovell's studies, the baseline levels of LUCot were similar; however, in Hovell's data, average LUCot was stable over time, whereas in Wilson's study it decreased significantly in both groups and significantly more in the intervention group, i.e., there was evidence of a beneficial effect of the intervention on LUCot, which was the primary study outcome variable.

Evidence of trends in correlations within intervention arm was likewise mixed. We found significant trends only in the control group in Hovell's study and in the control group in Wilson's study. In Hovell, the correlation between NSMOKERS and LUCot increased significantly over time (p=0.0078). The correlations between LANic and NCIGS and between LANic and NCIGSHOME decreased significantly over time (NICGS: p=0.0371, NCIGSHOME:

p=0.0307). In Wilson, the correlation between NSMOKERS and LUCot increased significantly over time (p=0.008).

© 2022 Bitan M. et al.
